# Unlocking sea turtle diving behaviour from low-temporal resolution time-depth recorders

**DOI:** 10.1038/s41598-025-05336-y

**Published:** 2025-06-06

**Authors:** Jessica Harvey-Carroll, Javier Menéndez-Blázquez, Jose Luis Crespo-Picazo, Ricardo Sagarminaga, David March

**Affiliations:** 1 Department of Biological and Environmental Sciences, University of Gothenburg, Gothenburg, Sweden; 2 Gothenburg Global Biodiversity Centre, Gothenburg, Sweden; 3 Cavanilles Institute of Biodiversity and Evolutionary Biology, Universitat de València, Valencia, Spain; 4 Ciudad de las Artes y las Ciencias, Fundación Oceanogràfic de la Comunitat Valenciana, Valencia, Spain; 5 Alnitak, Madrid, Spain; 6 Centre for Ecology and Conservation, College of Life and Environmental Science, University of Exeter, TR10 9 FE Penryn (Cornwall), Devon, UK

**Keywords:** Telemetry, Loggerhead turtle, Dive analysis, Seasonal behaviour, Hidden Markov model, Behavioural ecology, Animal behaviour, Ecology, Animal migration

## Abstract

Biologging is a rapidly advancing field providing information on previously unexplored aspects of animal ecology, including the vertical movement dimension. Understanding vertical behaviour through the use of time-depth recorders (TDRs) in marine vertebrates is critical to aid conservation and management decisions. However, using TDRs can be particularly problematic to infer animal behaviour from elusive animals, when tags are difficult to recover and collected data is satellite-relayed at lower temporal frequencies. Here, we present a novel method to process low-resolution TDR data at 5-minute intervals and infer diving behaviour from loggerhead turtles (*Caretta caretta*) during their elusive pelagic life stage spanning extended periods (> 250 days). Using a Hidden Markov Model (HMM) we identify four behavioural states, associated with resting, foraging, shallow exploration, and deep exploration. Three of the four behavioural states were found to have strong seasonal patterns, corroborating with known sea-turtle biology. The results presented provide a novel way of interpreting low-resolution TDR data and provide a unique insight into sea turtle ecology.

**Supplementary Information:**

The online version contains supplementary material available at 10.1038/s41598-025-05336-y.

## Introduction

Understanding the movement of animals is fundamental for increasing knowledge of basic biology and ecology^1^. This knowledge can then be used to aid conservation and protection of species and ecosystems^2–4^. Biologging is a rapidly developing technology to provide new insights into the elusive lives of marine animals^5^. A relevant insight is the vertical component of animal ecology, which up until recently has been neglected due to technological limitations such as hardware and software development^6^. Vertical dimensions provide otherwise unobtainable information on behaviour and survival, which is critical for understanding aquatic species^7^. In addition to novel insights, vertical information can complement other methodologies used in ecology, for example improving abundance estimates from survey data^8^. Inclusion of vertical information in management decisions has ample potential to improve targeted conservation measures such as vessel strike and bycatch risk assessment^2,7,9–14^. Despite the importance of vertical movements, they are considerably less understood than horizontal movements^15,16^.

Globally, sea-turtle species are of conservation concern, and lack of fundamental information on ecology limits conservation efforts^17,18^. Populations are rapidly declining due to anthropogenic threats such as bycatch, habitat loss and overharvesting^19^. Studying diving behaviour can provide important information on physiology and ecology, which in turn can help develop understanding of overlap with fisheries and mortality associated with bycatch^20,21^. One such way to study the vertical behaviour of sea-turtles is through time-depth recorders (TDRs). TDRs are a popular type of sensor that can be incorporated into satellite tags for measuring the vertical movements of marine animals^22^. Like most other forms of biologging, a trade off must be made between data resolution, transmission capacity and battery duration^22–24^. High resolution data typically requires manual retrieval of the device, which is challenging for elusive species or life stages. Alternatively, data may be compressed on board the devices, with dive summaries sent remotely through a constellation of satellites (e.g. Argos). The compression may lead to some elements of data loss^22,23,25–27^. Such trade-offs lend favour to devices which can transmit lower resolution data, overcoming the issues of animal recapture, or data loss. Because of these limitations, satellite devices, which are capable of transmitting customized highly accurate information on depth and location have become commonplace for studying air-breathing marine vertebrates, such as sea-turtles^6,28,29^. TDR satellite tags come at a high financial cost, increasing the scarcity of long-term datasets^29^.

The complexity of movement data acquired by continuously advancing technology has been met with the demand of using complex statistical methods^30–32^. Until recently, the most common method of interpreting TDR readouts has been through initially visual, followed by automated reconstruction (e.g., through cluster analysis and discriminant functions) of dive profiles^33^. Multiple dive profiles have been categorized and identified in pinnipeds, seabirds and turtles which may be used to infer behaviour^34,35^. However, the exact behaviours underlying dive profiles of many vertebrates, including sea turtles, remains largely debated^35,36^.

Hidden Markov models (HMMs) are increasingly popular for use in telemetry analysis given their capability of inferring underlying behavioural states, and transition probabilities between states, from multi-variate noisy observations of complex time-series data^37–40^. HMMs provide an alternative interpretation of TDR data, moving away from discrete dive profiles, and focusing on the behavioural ‘state’ of the animal. HMM’s have been used successfully to identify behavioural states from diving data for a range of air breathing vertebrates^38,41–44^. To date, the successful use of HMMs to infer behavioural states have used high resolution dive data (spanning seconds to one minute)^41,42,44^ or have focused on horizontal displacement only^45,46^. In this study we use a HMM to identify hidden behavioural states of 28 loggerhead turtles (*Caretta caretta*) from low resolution (5-minute) TDR data during extended periods (up to 292 days) across their elusive pelagic life stage.

## Methods

### Study site and data collection

Loggerhead turtles (*n* = 28) were tagged with satellite transmitters in the Western Mediterranean from 2015 to 2023, with a major focus on targeting juvenile or sub-adult individuals during summer months (Supplementary Tables 2 and Supplementary Fig. 1. Turtles were accessed from a recovery center (Palma Aquarium, *n* = 6), or captured during boat-based surveys on board of the RV Toftevaag (*n* = 22). During boat surveys, turtles were captured by hand as they rested at the surface by an observer swimming from an inflatable boat^47^. The midline curved carapace length (CCL) was measured for each turtle to the nearest 0.5 cm using a flexible tape. The carapace was first cleaned of biota with acetone and then lightly sandpapered to provide a better surface for transmitter attachment. All turtles were equipped with time-temperature-depth-recorders (TTDR) and ARGOS platform terminal transmitters (SPLASH10 tags, Wildlife Computers). TTDRs were programmed to collect depth (0.5 m resolution, ± 1% accuracy of the reading) and temperature (0.05 ºC resolution, ± 0.1ºC accuracy) and relay data via satellite in the format of continuous records at 5-min intervals. Due to the compression algorithm, resolution of data was determined by the dynamic range of the depth readings during the 5-minute period (K Lay, Wildlife Computers, personal communication). Transmitters were covered in MICRON 66 antifouling paint and attached to the carapace using a two-part epoxy resin (Wildlife Computers attachment kit). Tags were attached with the antenna facing forward along the turtle’s first and second vertebral scutes, so that the wet/dry sensor was out of the water when the animal surfaces. The tag attachment process took around 2 h to complete and then the turtles were released at the site of capture. Transmitters represented < 2% of the turtles’ body mass.

All tagging was conducted under authorisations from the Spanish Government through the “Ministerio de Medio Ambiente y Transición Ecológica” (Refeferences DIV/BDM/AUTSSP/58/2015 and SGBTM/BDM/AUTSPP/19/2022).

### Processing tracking data

Near-duplicate positions, defined as animal positions that occurred 2 min or less after an existing position fix from the same animal, were removed^48,49^. Argos data were then filtered using a speed, distance and angle filter^50^ that removed all location class Z values and points with unrealistic swimming speeds (> 5 km h^−1^)^51^ or unlikely turning angles (all spikes with angles smaller than 15 or 25 degrees were removed if their lengths were greater than 2.5–5 km, respectively) using the “argosfilter” R package^50^. Tracks with data gaps in excess of 7 days were trimmed into different trips (i.e. each portion of the track was treated independently). A state-space model (SSM) with a correlated random walk model was used to estimate locations at regular time intervals (2 h) and account for measurement error in the original observations using the “aniMotum” R package^52^. A move persistence model (MPM) was applied to the SSM-estimated locations^53^. This produced an index (*g*) of movement behaviour, representing changes in movement pattern based on autocorrelation in speed and direction in a continuous scale (0–1). Move persistence (MP) was reclassified into two classes based on the median threshold value of *g* ($\:\stackrel{\sim}{g}$) for each individual. MP’s with a value of g < $\:\stackrel{\sim}{g}$ represent localised movement, whereas g ≥ $\:\stackrel{\sim}{g}$ represent transiting movements^52^. All models were checked for convergence.

### Processing TDR data

**Fig. 1:**
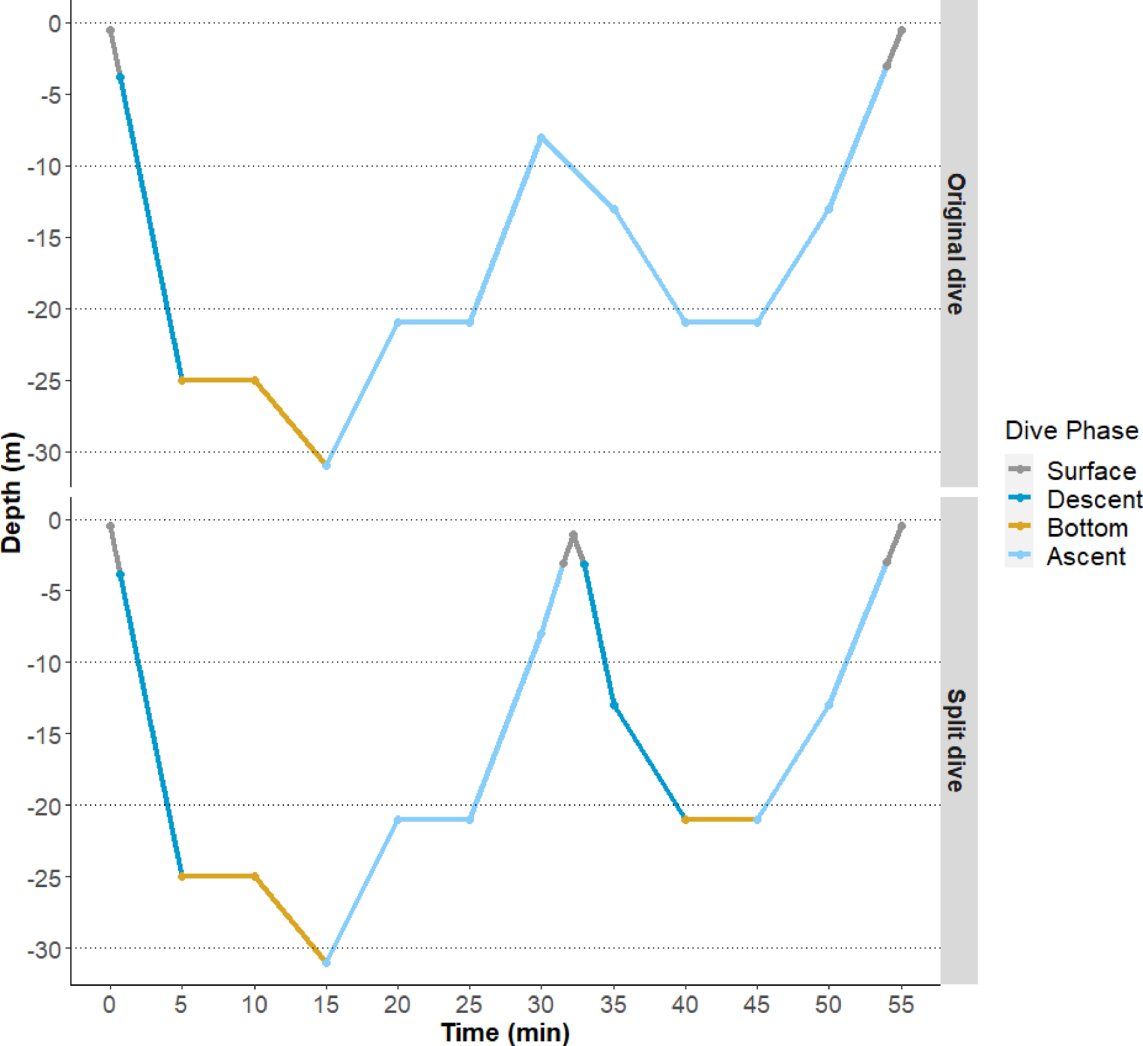
Depiction of dive profile automatic phase labelling and segmentation. Bottom phase is labelled as depth > 80% maximum depth. If peaks were detected (top panel), and it was possible the turtle could have surfaced given previous ascent speed a single dive the dive was ‘split’, creating two consecutive dives (bottom panel).

The SPLASH tags transmitted depth recordings at 5-minute intervals. First, TDR data was zero-offset corrected in order to account for depth sensor drift using the “diveMove” R package^54,55^. To define a dive, we used a dive threshold of 3 m based on previous studies^8,56,57^ and any depth readings below this depth were labelled as ‘dive’. The time at which the individual turtle first crossed the threshold value was calculated for each dive using a linear interpolation with intervals of 10 s. This was done by using the ‘aspline’ function from the “akima” R package^58^. This interpolation step was repeated to calculate the end of ascent, using the last reading for each dive below the threshold. If missing depth readings were present within dives (i.e. due to data gaps in satellite-relayed information) then these dives were excluded. The descent, bottom and ascent phases of dives were assigned. The bottom phase was determined to be any depth over 80% of the maximum dive depth^59,60^ (Fig. 1). Depths below 3 m preceding the bottom phase were assigned ‘descent’. Depths following the ‘bottom phase’ prior to reaching the 3 m threshold were assigned ‘ascent’.

### Dive segmentation

Low resolution of the TDR data may cause fast successive dives to appear as a single longer dive, resulting in the occurrence of ‘peaks’ in depth. The peaks occur when the turtle ascends above 80% of the maximum depth and then rapidly descends again, likely because the ascension and descension of the turtle past the dive threshold were not accurately recorded. Peaks over 1 m within dives were detected using the R package “pracma”^61^. After detection of peaks, two potential times at which the turtle would have ascended past the 3 m dive threshold were determined. The first time was based on the previous speed of the turtle between readings, and the second time was based on the future speed of the turtle between readings. If either the rate of ascension or descension was sufficient for the turtle to surface within the time interval before the next reading, the dive was split into two successive dives (Fig. 1). Such splitting was also conducted for dives where a turtle ascends, remains at a given depth and then descends, without surfacing.

### Validation of method using high-resolution TDR datase

To assess the dive segmentation approach of low-resolution dives, and validate the aforementioned data processing, we used a high-resolution dataset (1 s depth readings) obtained from a recovered Survivorship PAT (sPAT, Wildlife Computers) deployed on a loggerhead turtle (Supplementary Table 1) from the south Adriatic Sea to study post-release mortality in trawling fisheries (Crespo et al. unpublished data). The dataset spanned 12 days starting 30 th January. TDR data was then resampled at 300 s and processed for both 1 s and 300 s sampling frequencies as described in the methods section (Sect. 2.3). Maximum depths were then obtained for each dive. Frequency of dives were pooled by their maximum depth in 10 m intervals. Fishers exact test was conducted to assess the difference in frequencies of the maximum depths between the 1 s and 300 s sampling intervals.

A total of 251 dives was found for the one second sampling, whilst a total of 109 dives was identified for the resampled 300 s interval. Dives below 10 m were excluded from analysis due to the known occurrence of rapid dives below 10 m. A total of 82 dives were identified below 10 m for the total duration of deployment for the one second sampling interval. For the five-minute sampling, a total of 60 dives were identified. Fishers exact test found no significant difference between maximum depths of dives (*p* = 0.77, Supplementary Fig. 1).

As result of such validation, all dives with a maximum depth equal to, or below 10 m were excluded from further analysis due to the low resolution (Supplementary Fig. 2)^35^.

### Multivariate hidden markov model (HMM)

To infer behavioural states for each dive, we used a multivariate Hidden Markov model (HMM). First, dives within the first 24 h of deployment were excluded from further analysis to conservatively minimize the potential impact of animal handling on sea turtle behaviour^15,44^. Then, four dive variables were extracted for each dive: maximum depth, bottom time (i.e., time spent over 80% of maximum dive depth), dive time and post-dive surface interval (i.e. the time elapsed between the end of the ascent and the following dive). Dive duration and maximum depth are common descriptors of diving behaviour in marine vertebrates, and represent the effort expended during a dive^62,63^. We also included post-dive surface interval as it has been implicated as an indicator of metabolic costs following a dive^64^. Additionally surface intervals have been associated with basking behaviour, and are negatively correlated with foraging search effort^63–65^. Due to the large differences in units used, data was normalized using the preprocessing option “range” from the “caret” package^66^ resulting in all variables scaled between zero and one. In addition, post-dive surface interval, maximum depth and dive time were log-transformed due to their skewed distributions.

HMMs were run for two, three, four and five states using the “depmixS4” R package^67^ and previous R code^43^. We did not exceed five states due to biological interpretability^68^. Each model was fit 100 times from random initialization to account for numerical stability and robustness^43^. The iteration with the lowest AIC score was retained for each model. AICs of the models were then compared (Supplementary Table 2). The output of the best fitting model for each number of states were assessed. Using biological knowledge to guide state selection over use of a formal model selecting procedure has been done successfully for diving mammals^42,69^. The model consisting of four states was assessed to be most appropriate, as the five-state model did not produce any additional biologically distinct states and featured considerable overlap with pre-existing states. To assess differences in proportions of g < $\:\stackrel{\sim}{g}$ and g ≥ $\:\stackrel{\sim}{g}$ across HMM states, a binomial generalised linear model was conducted^70^. MPM class (localised, g < $\:\stackrel{\sim}{g}$ or transiting, g ≥ $\:\stackrel{\sim}{g}$) was defined as the dependant variable, with HMM state as a main effect. Following significance a Tukey pairwise comparison was conducted.

### Temporal dive analysis

To investigate seasonality of states identified from the HMM, the number of dives performed in each state per month for each turtle was calculated. Next, generalized additive mixed models (GAMMs) were conducted using the R package “mgcv”^71^. We fitted cyclic cubic regression splines to ensure that intercepts at month 1 (January) and 12 (December) aligned, in keeping with the circular nature of the data, and adjusted the knots in the spline to avoid over- or underfitting^72^. One model was conducted for each state. Models were built using the binomial distribution with logit link, and conducted for proportion of dives in State 1, State 2, State 3 and State 4 (as opposed to all other categories), respectively^73^. Individuals were added as random effects. No significant temporal autocorrelation was identified for State 1, 3 and 4 models using ACF and PACF plots from the “tseries” R package^74^. For the state 2 model significant autocorrelation was detected. For this state, a GAMM was conducted with inclusion of a first order autoregressive correlation term to account for autocorrelation of residuals. We present summaries and visualizations for models fit using REML^72^ and 6 knots because those models were not unduly affected by low numbers of atypical observations during poorly sampled periods.

### Data and code availability

All analysis and plots were undertaken using the R programming language^75^. The data and code are available at: 10.5281/zenodo.15582972.

## Results

### Telemetry data

From the deployments across 28 turtles, a total of 4,835 days of dive data at a 5-minute resolution was obtained. The mean duration per deployment was 173 days (range 13–292 days). A total of 42,618 dives below 10 m depth were obtained (Table 1). A mean of 1,522 dives were obtained per deployment (range 39 − 3,987 dives). The deepest dive recorded was 313.5 m (upper depth error: 343.9, lower depth error: 283.62). The longest recorded bottom time was 235 min, the longest total dive time was 290 min and finally the longest post-dive interval (surface time) was 27.5 h. Two turtles ventured into distant regions during their deployments: one travelled from the west to the east Mediterranean, nearing the coast of North Africa, while another reached the West African coast in the Atlantic.

### State identification

**Fig. 2:**
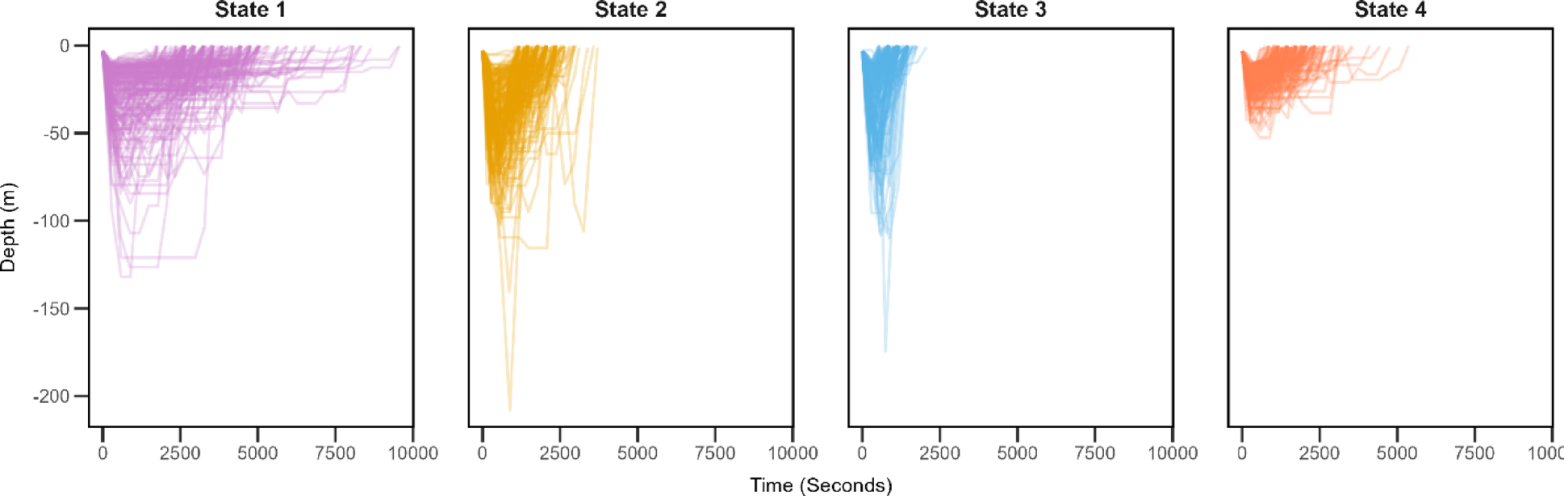
Hidden Markov models suggested four behavioural states. A random selection of 200 dives per state are shown. Surface interval is not depicted.

**Fig. 3:**
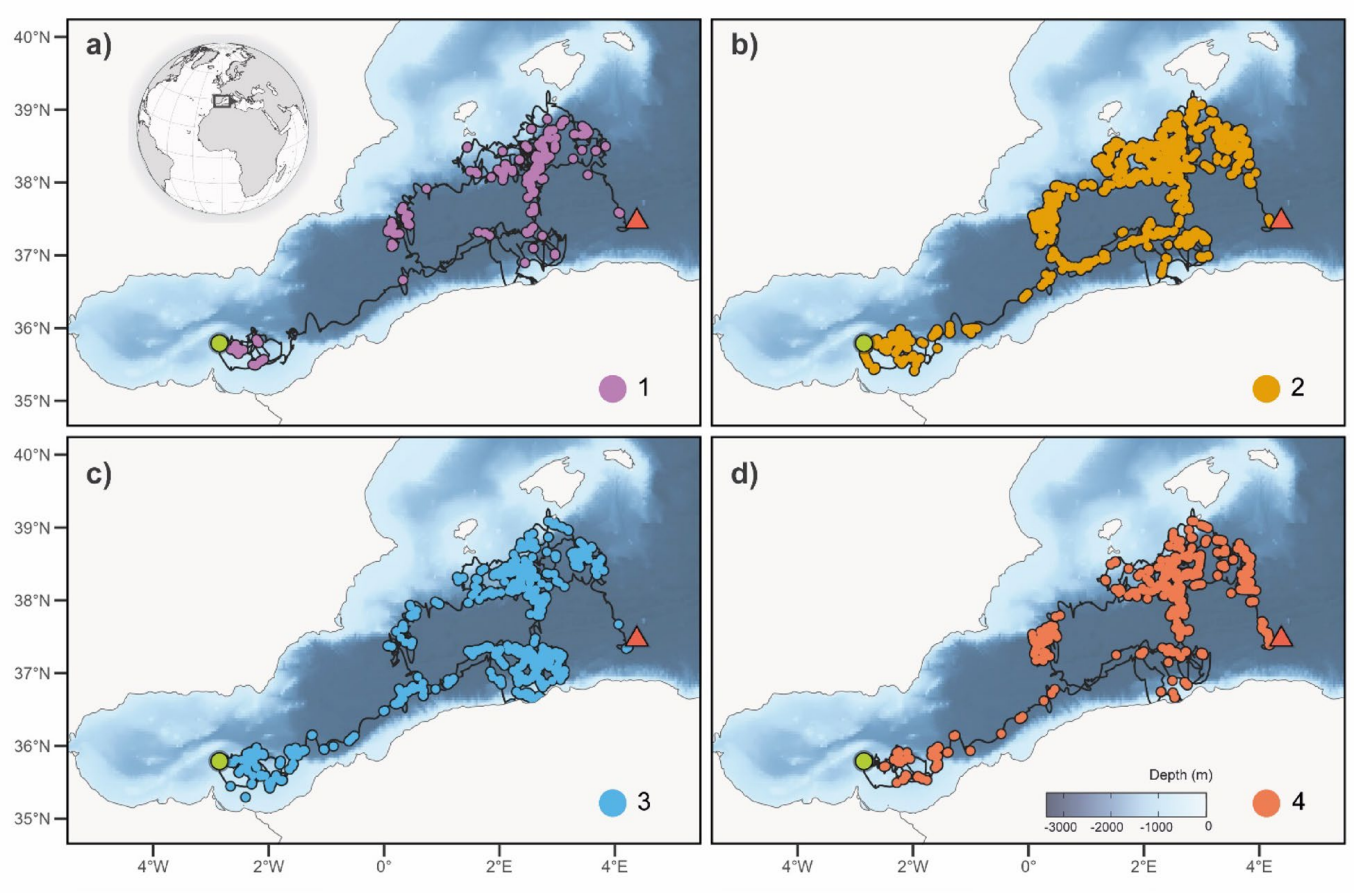
Location of each depicting behavioural state throughout single TDR deployment (organism ID 235396; data spanning 214 days): (a) State 1 = Purple, (b) State 2 = Orange, (c) State 3 = Blue and (d) State 4 = Coral. (b) Location of each behavioural state throughout. deployment; Green dot and red triangle showing the beginning and the ending of the track visualized, respectively.

Four behavioural states were identified from the HMM representing different dive profile types (Figs. 2 and 3, Supplementary Figure X).

State 1 featured dives with an intermediate maximum depth. The longest bottom time was identified alongside the longest dive time. A short surface interval was also identified. State 2 was characterized by the deepest maximum depth and an intermediate bottom time. An intermediate dive time was identified, followed by a long surface interval. For State 3, an intermediate maximum depth was identified, with the least bottom time. State 3 featured the shortest dive time, proceeded by the longest surface interval. Finally, State 4 had the shallowest maximum depth with an intermediate time spent there. State 4 had an intermediate dive time, followed by the shortest surface interval. Individual dive variables featured overlapping ranges (Supplementary Table 3), however when considering multiple variables together overlap of individual variables was reduced considerably (Supplementary Fig. 4).

**Fig. 4:**
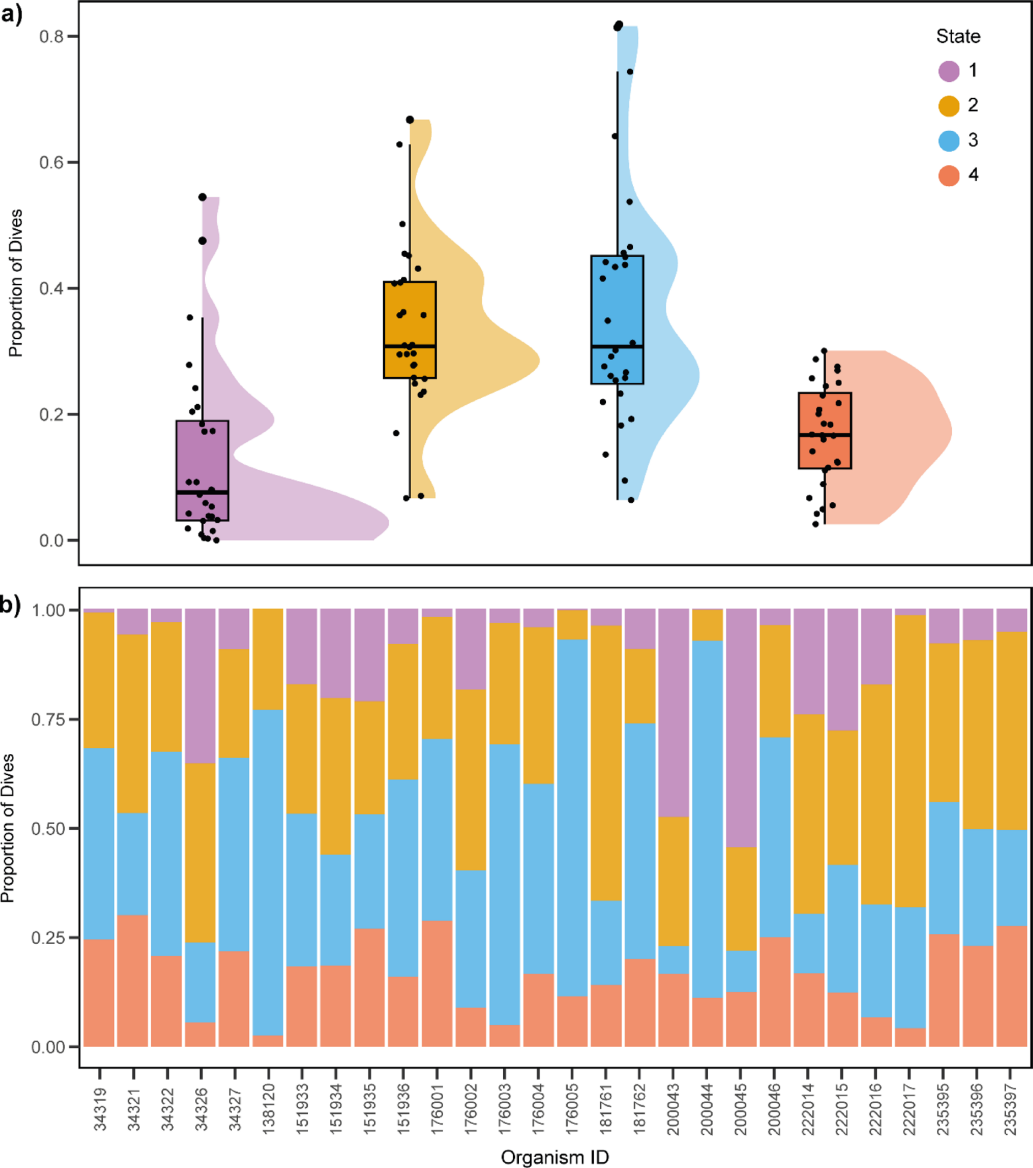
State allocations across all loggerhead turtles. (**a**) Proportion of dives in each state across all individuals. Boxplots depict median relative expression levels and the 25 th and 75 th percentiles. Whiskers are 1.5× the interquartile range, data points outside this range are marked as outliers (circles). (**b**) Proportion of time each turtle spent in each state for the entire deployment duration. Turtle IDs correspond to Argos PTT numbers.

States 2 and 3 occurred most frequently within our dataset (Fig. 4a). Similarly, individuals generally spent the highest proportion of time in States 2 and 3. The least amount of time was spent in state 4. The time spent in State 1 appeared to feature some individual variation; 18 individuals spent less than 1% of their time in it, while two spent more than 47% (Fig. 4b). Maps of all individuals and states can be seen in Supplementary Fig. 5.

### State transitions

All states were found to be stable with a low probability of transitioning to a different state (Table 1; Fig. 4A, B). Individuals had the highest probability of starting in State 3 (0.601) compared to State 4 (0.270), State 2 (0.081), or State 1 (0.048).

**Table 1:** State transition probabilities.

	To state 1	To state 2	To state 3	To state 4
From state 1	0.833	0.042	0.052	0.072
From state 2	0.025	0.804	0.104	0.068
From state 3	0.023	0.108	0.781	0.088
From state 4	0.072	0.112	0.163	0.652

### Move persistence

**Fig. 5:**
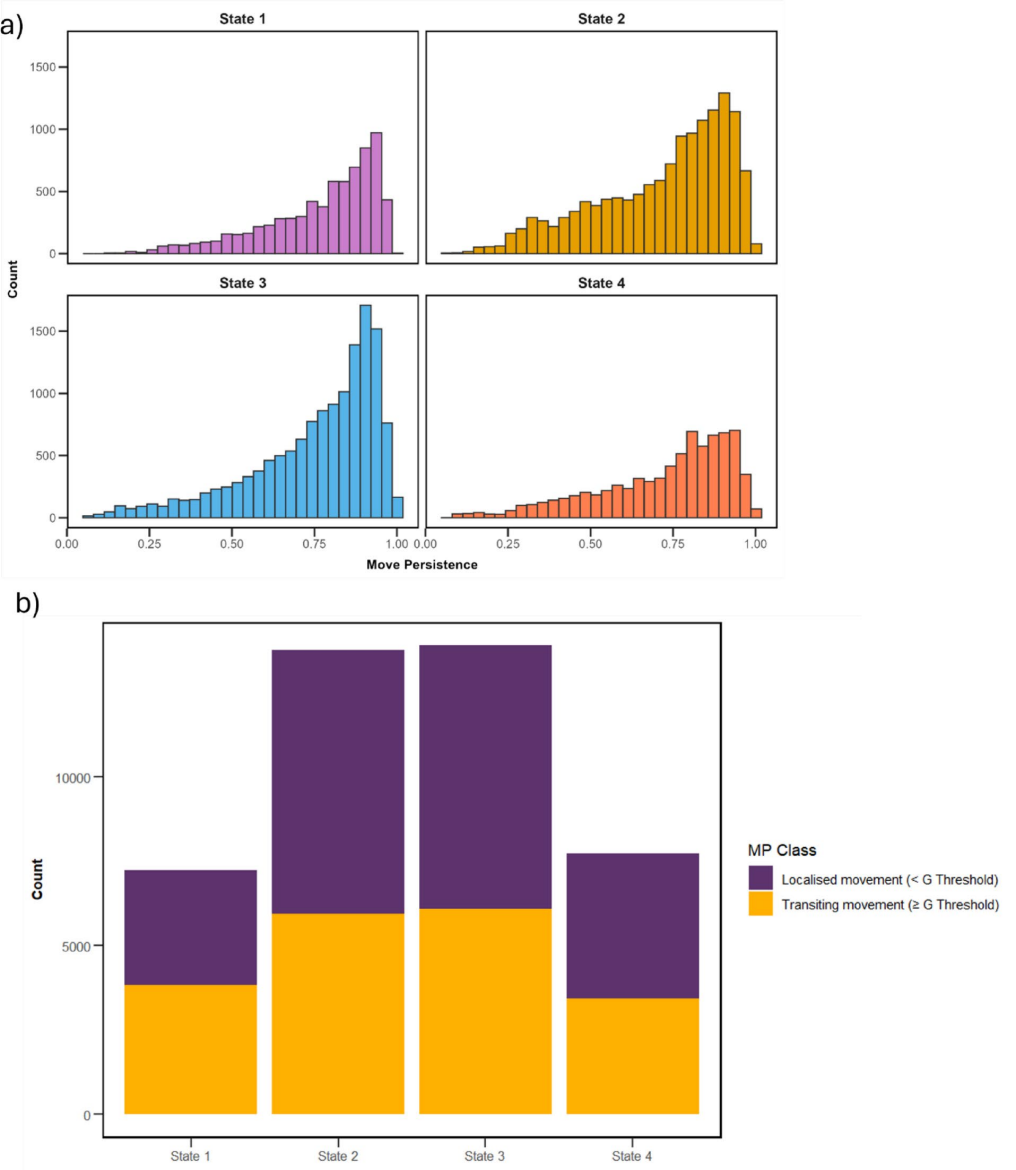
Move persistence metrics across all deployments. (**a**) Histograms of move persistence metric for each identified state for all dives across all individuals. (**b**) Proportions of MP classes within each identified state.

Regardless of state, high levels of move persistence (MP) between individual dives were found across all turtles (Mean: 0.74, SD: 0.20). Relatively high MP’s were present within each state (State 1 Mean: 0.77 SD: 0.17; State 2 Mean: 0.72 SD: 0.20; State 3 Mean: 0.75 SD:0.19; State 4 Mean: 0.73 SD: 0.20. Figure 5a). State 1 exhibited a significantly lower proportion of ‘localised’ movement (g < $\:\stackrel{\sim}{g}$) when compared to states 2,3 and 4 (Table 2, Supplementary Table 4, Fig. 5b). Maps of all individuals and MPs can be seen in Supplementary Fig. 6.

**Table 2:** Pair-wise comparisons of transitory and localized movement proportions between states.

Comparison	Estimate	SE	Z ratio	*P* value
State 1 – state 2	0.396	0.029	13.568	< 0.0001*
State 1 - state 3	0.372	0.029	12.787	< 0.0001*
State 1 - state 4	0.342	0.033	10.431	< 0.0001*
State 2- state3	−0.024	0.024	−0.975	0.764
State 2- state 4	−0.053	0.029	−1.860	0.245
State 3 - state 4	−0.030	0.029	−1.037	0.728

### Seasonality

**Fig. 6:**
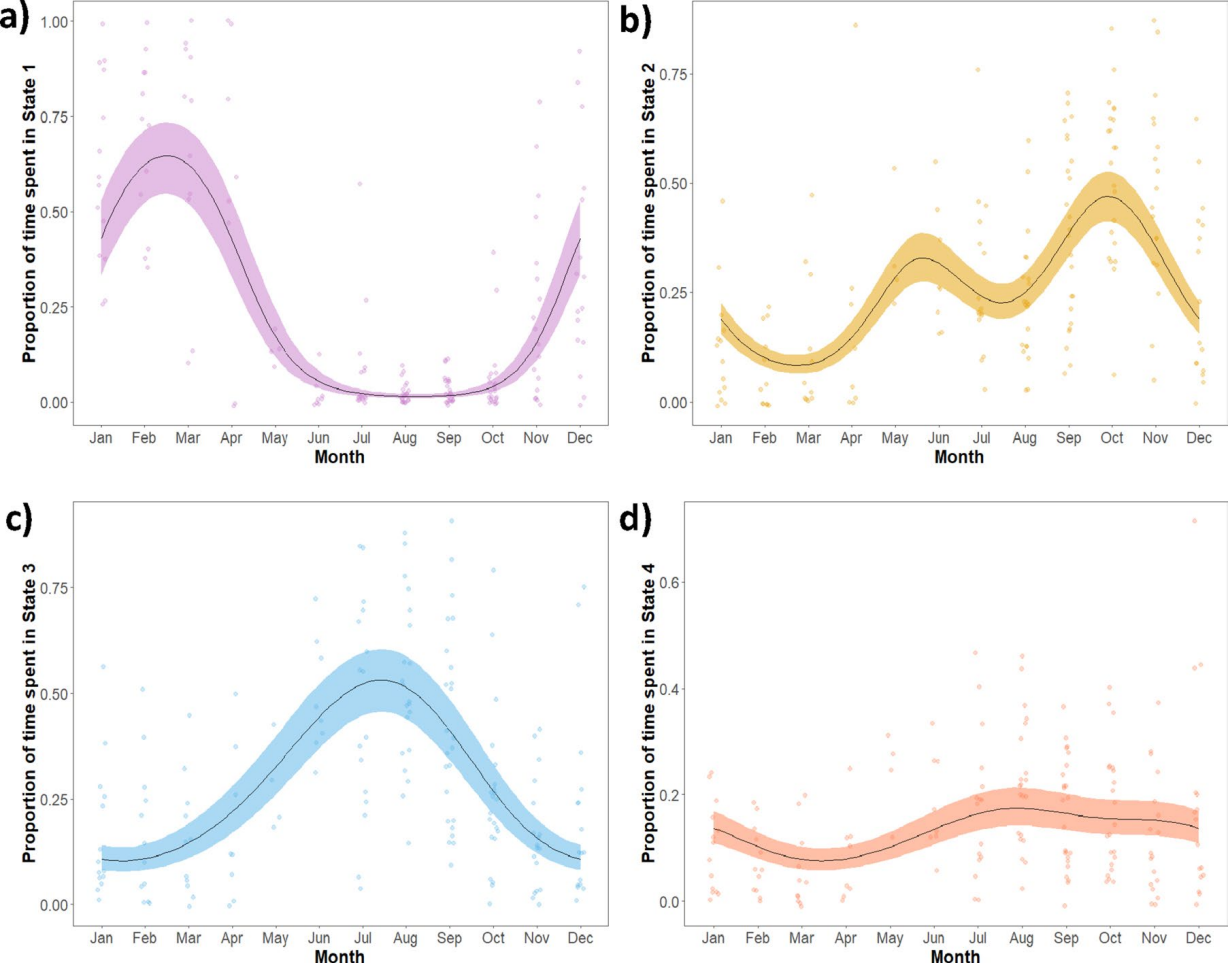
Plots of best fitting GAMMs to assess monthly variation of the time spent in each of the four behavioural states across loggerhead turtles.

A significant smoothing effect of month on proportion of time spent in each state was identified (Table 3; Fig. 6). GAMMs suggested strong seasonal patterns for proportion of time spent in States 1 and 3, which present opposite responses, with higher proportion of State 3 during summer. State 2 appeared to feature a seasonal pattern with two main peaks. State 4 featured a slight decrease in proportions during the spring months. The patterns were not influenced heavily by method of spline fitting or number of knots used.

**Table 3:** Results of the best-fitting gamm’s for proportion of time spent in each state.

	Estimate	Std. Error	z value (State 2 t value)	Pr(>|z|)
**State 1**				
(Intercept)	−2.15	0.20	−10.58	< 0.00001
Approximate significance of smooth terms:				
	edf	Ref.df	Chi.sq	p-value
Month	3.97	4	632,479	< 0.00001
Individual	25.22	27	3531	< 0.00001
**State 2**				
(Intercept)	−1.07	0.12	−9.23	< 0.00001
Approximate significance of smooth terms:				
	edf	Ref.df	Chi.sq	p-value
Month	3.98	4	3570.91	< 0.00001
Individual	26.25	27	51.85	< 0.00001
**State 3**				
(Intercept)	−1.04	0.15	−6.84	< 0.00001
Approximate significance of smooth terms:				
	edf	Ref.df	Chi.sq	p-value
Month	3.94	4	120,264	< 0.00001
Individual	26.531	27	3792	< 0.00001
**State 4**				
(Intercept)	−1.82	0.13	−14.54	< 0.00001
Approximate significance of smooth terms:				
	edf	Ref.df	Chi.sq	p-value
Month	3.81	4	2350	< 0.00001
Individual	25.86	27	1206	< 0.00001

## Discussion

In this paper, we use a long-term TDR dataset of tracked loggerhead turtles to demonstrate that low resolution (5-minute) sampling can be used to reconstruct dive profiles and identify diving behavioural states. The long duration of deployments enabled multiple observations of individuals across months and seasons. We present the first use of HMM’s to infer behavioural states from dive information alone in sea turtles. This automated approach offers a reproducible alternative to visual dive labeling of large scale datasets, negating issues such as time required and potential human error.

While some studies have successfully deployed long-term tags that retain high-resolution data (1–15 s intervals^76–78^), these typically require physical retrieval, which can constrain sample size and spatial coverage. In contrast, the remotely transmitted, low-resolution data used in our study allow for broader deployment and long-term tracking without the need for tag recovery. Throughout the deployments the deepest recorded depth was 314.5 m (upper depth error: 343.9 m, lower depth error: 283.62), from a 66 cm CCL turtle. This falls within the range of previously recorded maximum dive depths exceeding 340 m (from two male North Pacific loggerhead turtles (SCL 77.8 & 82.5 cm))^79^.

Using short-term, high-resolution TDR data, previous works on loggerhead turtles have identified eight dive profiles^35^. Classification of dive profiles has previously been conducted visually^80–82^. Dive profiles may, however, overlap in behavioural states due to limited validation of specific behaviours within each profile or true overlap of characteristics between profiles. For example some dive profiles have been attributed to travelling, whilst the same have also been attributed to mid water resting^35,83^. Focusing instead on behavioural states derived from observable metrics may mitigate the aforementioned issue. For example, dive summary metrics from onboard tag processing have previously been combined with horizontal movement in changepoint analysis to identify behavioural states such as migration, foraging and overwintering in loggerhead turtles^32^. Additionally, through employment of a HMM, three behavioural states (transit, high and low intensity diving) have been identified in loggerhead turtles. The states were produced by combining number of dives performed and horizontal movement from high-resolution tags^44^.

From the use of derived dive summary metrics with HMM’s we identified four distinct behavioural states, three of which showed strong seasonality. The states may be attributed to ecologically relevant behaviours using known characteristics for diving of sea turtles from previous studies^35,64,83,84^. In short, we identified behavioural states which may be inferred as resting, mid-water foraging and finally two types of exploratory/transit dives. As with all HMM approaches, validation using ground truthing is required to confirm inferences made^85^. The seasonality identified in these states corresponds to previously identified seasonal trends in sea turtle diving behaviour, lending support to behavioural inferences^35,80,84,86,87^.

State 1 consisted of the greatest time spent over 80% if the maximum depth and consequently the longest overall dive time. A short surface interval following this was present suggesting that the dives were not metabolically expensive^64,86^. Given these characteristics combined with seasonal prevalence during colder months, this state likely represents ‘resting dives’ (previously referred to as Type A dives which feature a U-shaped dive profile)^35,80,84,86^. From these characteristics it may be inferred this state is comparable to the overwintering or brumation dives previously identified in sea turtles, in which a large proportion of the total dive time is spent at maximum depth, likely resting. In this state, reduced activity and lower metabolic rate likely occur^86^. Additional support that State 1 represents ‘resting dives’ comes from the identified temporal clustering (as seen by the high transition probability). This may represent the sequential (continuous) resting previously identified in green and loggerhead turtles^83,86,88–90^.

State 2 consists of the deepest dives. In this state, only a small amount of time was spent > 80% of the maximum dive depth, suggesting fast movement. Because of this, it likely represents deeper exploratory behaviour, frequently seen during transit in juvenile loggerhead turtles^91^. This may be likened to dive profiles referred as Type C (which present as a V-shaped dive)^35^. However, unless ground-truthed it is not possible to exclude the possibility of foraging. The peak expression of this state was seen in October, with the lowest expression during February and March.

State 3 featured a long dive time, with the least amount of time spent at depths > 80% maximum depth. This state was found to have the longest surface interval across all states. When combined these suggest the majority of activity is likely occurring during the ascent phase, similar to a type E (S-shaped, after reaching the maximum depth a sharp ascent to a specific depth occurs, followed by a slow ascent, and a final steep ascent to surface)^35,90^ or F dive (with a gradual ascent from maximum depth with a steep final surface ascent)^35,92^. As such, this may be indicative of mid-water foraging or travelling behaviours. The characteristics of this dive correspond to previously identified mid-water foraging dives identified^93^. Furthermore, the long surface duration proceeding a shorter dive time may indicate foraging (high search effort) during the dive^64^. A seasonal peak in summer months was identified, supporting the inference of summer foraging behaviour^35^. Without ground-truthing of this state it can only be ascertained that a large degree of mid-water activity is present.

Both states 2 and 3 feature long post-dive surface intervals. As peak expression of these states occurs during October (State 2) and July/August (State 3) it is difficult to distinguish the function of this behaviour relating to basking or high metabolic activity during a dive^64,65,94^. There is currently conflicting evidence in sea turtles on the relationship to basking and temperature, for example green turtles have been found to increase basking time with decreasing SST^95^. Western Mediterranean juvenile loggerheads have been found to spend more time at the surface in spring, but not in summer months as the increased SST does not necessitate basking^96^. Conversely, juvenile loggerheads in the Atlantic ocean have been found to spend more time at the surface during spring and summer (March-August)^64^. Given the higher SST temperatures in July/August within the Mediterranean it may be likely that the longer post-dive surface intervals seen in State 3 are associated with high metabolic dive activity.

Finally, State 4 consisted of the shallowest maximum depth and the shortest surface interval. This suggests that state 4 dives are less metabolically costly dives and can likely be attributed to exploration dives, or shallow mid-water foraging as seen in previous studies^83,93^. This state featured the least seasonal variation. The persistence of these dives regardless of season suggests they are an important aspect of loggerhead turtle behavioural ecology.

The states identified in this paper appear to be loosely comparable to the three diving behaviours identified in juvenile loggerheads using depth and duration^62^. That is, two variations of shallower dives (States 1 and 3) and finally deeper dives of a longer duration (State 2). It is important to note multiple behaviours may occur simultaneously (e.g. opportunistic predation whilst undertaking an alternative dive). Regardless of the true behavioural underpinnings, the information obtained from the identified states still provide novel information into the ecology of a cryptic species. For the states presented, individual variables featured a large degree of overlap between dives. However, when multiple variables were considered together, clear groupings were observed, with minimal overlap. This likely reflects the well-known multi-dimensional complex nature of dives in marine vertebrates^97^. The need for integration of multiple variables to infer behavioural states has previously been identified in short-finned pilot whales, and represents the complexity of diving behaviour^41^. HMM’s allows for identification of temporal clustering of behaviours within dive times series^38^. Temporal clustering of behaviour was identified, as indicated by the high stability of states. This suggests that states are more likely to occur successively. This confers with previous suggestions of sea turtle diving patterns^83^. Furthermore, the temporal clustering of states appears to be common in other air-breathing marine vertebrates such as whales^41,43,98^.

Discrepancies between temporal frequencies of TDR readings (5 min) and SSM (2 h) occurred for the data used in this study. Such discrepancies may explain the differences observed between horizontal and vertical movements (e.g. State 1 was characterised as resting behaviour, and yet had the lowest proportions of localised horizontal movements across states). MP was estimated at two-hour intervals. Higher levels of move persistence were identified across all behavioural states, and featured a large degree of overlap. This may suggests that the juvenile loggerhead turtles in this study generally displayed more linear movements regardless of behavioural state^53^. The overlap of MP across states supports the concept that addition of the vertical dimension is critical to inform the understanding of animal behaviour over longer periods, as horizontal movements alone do not reflect vertical movements^63^. Future work should be aimed towards an integration of both horizontal and vertical dimensions to provide a comprehensive assessment of marine organism behaviour.

One caveat of the data presented here, as suggested by the resampling of high frequency dives to a lower frequency, is the omission of fast movements. Furthermore, the low sampling intervals lack derived metrics such as speed SD which are better inferred from higher resolution data. It is likely that additional states consisting of dive times less than five minutes may be present, but not detected by the low frequency deployments. The use of higher resolution TDR data, or complimentary data sources such as accelerometers^99^ in the HMM may improve accuracy of predicted states. Accelerometery or animal borne-cameras may also be useful to corroborate the inferred behaviours^100,101^. New advances in communication systems, such as Kinesis and Iridium^102^ may in the future allow for higher transmission rates and in turn higher resolution of data, such as TDR data, over long-term deployments.

Despite the identification of states, without further research using video loggers, or accelerometers the behavioural inferences from HMM states must be used with caution, as a true correspondence to biologically meaningful behaviour is not guaranteed^41^. This inference of behaviour from states is akin to the common practice of inferring behavioural states from dive profiles. The behavioural function of each dive profile has mixed evidence, possibly due to the reduced dimensionality of data from dive profiles alone^35,97,103^. Nonetheless, the states identified in this paper are relevant for loggerhead turtle conservation and protection, irrespective of their true behaviour. The characteristics of states corroborate previous knowledge of sea turtle behaviour such as extended bottom times and extended surface periods. The behavioural states presented in this paper can in turn be used to identify spatiotemporal ‘hotspots’ for bycatch and gas embolism risk, alongside collision risks with shipping^21,104,105^. Furthermore, quantifying the transitions between behavioural states may allow for assessment of the impacts of human activities on sea turtle behaviour. The methods presented in this paper may be used for analysis of low-resolution TDR data for other air-breathing marine vertebrates. This presents an option for deriving behavioural states relevant for understanding behavioural ecology from long-term deployments TDR deployments.

## Electronic supplementary material

Below is the link to the electronic supplementary material.

Supplementary Material 1

## Data Availability

The data and code are available at 10.5281/zenodo.15582972.
