# Supplementary material for: Unlocking sea turtle diving behaviour from low-temporal resolution time-depth recorders
Source: Sci Rep. 2025 Jun 6;15:19934. doi: 10.1038/s41598-025-05336-y (PMC12144214; doi:10.1038/s41598-025-05336-y)
Supplement: Supplementary file 1 — Supplementary Material 1 [file 41598_2025_5336_MOESM1_ESM.docx]

Supplementary Information

**Unlocking sea turtle diving behaviour from low-temporal resolution time-depth recorders**

Jessica Harvey-Carroll^1^, Javier Menéndez-Blázquez^2^, Jose Luis Crespo-Picazo^3^, Ricardo Sagarminaga^4^, David March^2,5^

^1^ Department of Biological and Environmental Sciences & Gothenburg Global Biodiversity Centre, University of Gothenburg, Sweden.

^2^ Cavanilles Institute of Biodiversity and Evolutionary Biology, Universitat de València, Valencia, Spain.

^3^ Fundación Oceanogràfic de la Comunitat Valenciana, Ciudad de las Artes y las Ciencias, Valencia, Spain.

^4^ Alnitak, Madrid, Spain.

^5^ Centre for Ecology and Conservation, College of Life and Environmental Science, University of Exeter, TR10 9FE Penryn (Cornwall), United Kingdom

Contents

1. Supplementary Tables (Supplementary Tables 1-4)

2. Supplementary Figures (Supplementary Figures 1-6)

1. Supplementary Tables (Supplementary Tables 1-4)

| Supplementary Table 1. Biometrics of and tracking information of loggerhead sea turtle tagged in this study. * Indicates high resolution deployment used in validation. | | | | | | |  |
| --- | --- | --- | --- | --- | --- | --- | --- |
| Organism ID | CCL | Sex | Age Class | Deployment Date | Duration of tracking (days) | Total dives recorded | Tag model type |
| 138120 | 50 | unknown | juvenile | 05-08-2015 | 25 | 39 | SPLASH10-296F |
| 151933 | 46 | unknown | juvenile | 09-07-2016 | 210 | 1286 | SPLASH10-F-344A |
| 151934 | 73 | unknown | juvenile | 26-07-2016 | 276 | 1210 | SPLASH10-F-344A |
| 151935 | 65 | unknown | juvenile | 21-06-2016 | 277 | 3277 | SPLASH10-296F |
| 151936 | 60 | unknown | juvenile | 26-06-2016 | 290 | 2293 | SPLASH10-296F |
| 176001 | 75 | unknown | juvenile | 01-08-2018 | 88 | 1723 | SPLASH10-344D |
| 176002 | 63 | unknown | juvenile | 30-07-2018 | 279 | 1752 | SPLASH10-344D |
| 176003 | 62 | unknown | juvenile | 01-06-2019 | 227 | 1932 | SPLASH10-344D |
| 176004 | 52 | unknown | juvenile | 30-07-2018 | 292 | 1556 | SPLASH10-344D |
| 176005 | 70 | male | juvenile | 10-05-2019 | 117 | 764 | SPLASH10-344D |
| 181761 | 61 | unknown | juvenile | 28-09-2019 | 13 | 78 | SPLASH10-344D |
| 181762 | 56 | unknown | juvenile | 11-07-2019 | 272 | 1865 | SPLASH10-344D |
| 200043 | 66 | female | juvenile | 09-07-2020 | 255 | 3381 | SPLASH10-344D |
| 200045 | 77 | female | adult | 31-07-2020 | 285 | 2765 | SPLASH10-344D |
| 34319 | 83 | female | adult | 21-06-2017 | 85 | 883 | SPLASH10-344D |
| 34321 | 66.5 | unknown | juvenile | 16-07-2017 | 86 | 915 | SPLASH10-344D |
| 34322 | 52 | unknown | juvenile | 03-07-2017 | 113 | 1042 | SPLASH10-344D |
| 34326 | 58 | male | juvenile | 26-08-2017 | 230 | 631 | SPLASH10-344D |
| 34327 | 57 | female | juvenile | 15-02-2018 | 288 | 1062 | SPLASH10-344D |
| 235395 | 65 | unknown | juvenile | 08-05-2023 | 70 | 1144 | SPLASH10-344D |
| 222014 | 71 | female | adult | 04-09-2021 | 149 | 1330 | SPLASH10-344D |
| 222015 | 55 | unknown | juvenile | 18-08-2021 | 199 | 2451 | SPLASH10-344D |
| 222016 | 57 | female | juvenile | 28-09-2021 | 213 | 1044 | SPLASH10-344D |
| 222017 | 55.5 | female | juvenile | 05-09-2021 | 62 | 475 | SPLASH10-344D |
| 200044 | 55 | unknown | juvenile | 06-06-2021 | 67 | 1108 | SPLASH10-344D |
| 235396 | 60 | unknown | juvenile | 08-05-2023 | 214 | 3987 | SPLASH10-344D |
| 235397 | 68.6 | male | juvenile | 31-07-2023 | 127 | 2305 | SPLASH10-344D |
| 200046 | 58 | female | juvenile | 08-06-2022 | 25 | 320 | SPLASH10-344D |
| 53319* | 54.5 | unknown | juvenile | 30-01-2018 | 12 | 82 | Axy Trek Marine |

| **Supplementary Table 2. Comparison of number of states used in HMM.** | | |
| --- | --- | --- |
| Number of States | AIC | Delta AIC |
| 2 | -273961 | 43997.13 |
| 3 | -294447 | 23511.01 |
| 4 | -307660 | 10298.41 |
| 5 | -317958 | 0 |

| **Supplementary Table 3. Mean and SD of response variables across 42618 dives labelled using HMM.** | | | | | | | | |
| --- | --- | --- | --- | --- | --- | --- | --- | --- |
|  | **State 1** | | **State 2** | | **State 3** | | **State 4** | |
| Response Variable | *Mean* | *SD* | *Mean* | *SD* | *Mean* | *SD* | *Mean* | *SD* |
| Maximum Depth (m) | 31.66 | 21.10 | 50.17 | 26.73 | 33.44 | 22.44 | 22.60 | 9.28 |
| Bottom Time (s) | 1,569.48 | 1,264.40 | 524.42 | 381.03 | 136.00 | 173.62 | 598.52 | 430.71 |
| Dive Time (s) | 3,906.92 | 1,544.45 | 1,861.71 | 515.89 | 1,079.72 | 358.11 | 1,751.21 | 682.27 |
| Surface Interval (s) | 1,199.65 | 4,499.52 | 2,204.12 | 5,809.49 | 2,567.58 | 5,935.64 | 56.49 | 22.92 |

| **Supplementary Table 4. Main effects output of binomial GLM for proportion of high vs low MP’s in each HMM state.** | | | | |
| --- | --- | --- | --- | --- |
|  | Estimate | Std. Error | z value | Pr(>\|z\|) |
| (Intercept) | 0.12274 | 0.02354 | 5.213 | 1.85E-07 |
| State 2 | -0.39571 | 0.02917 | -13.568 | < 2e-16 |
| State 3 | -0.37206 | 0.0291 | -12.787 | < 2e-16 |
| State 4 | -0.34245 | 0.03283 | -10.431 | < 2e-16 |

3. 2. Supplementary Figures (Supplementary Figures 1-5)


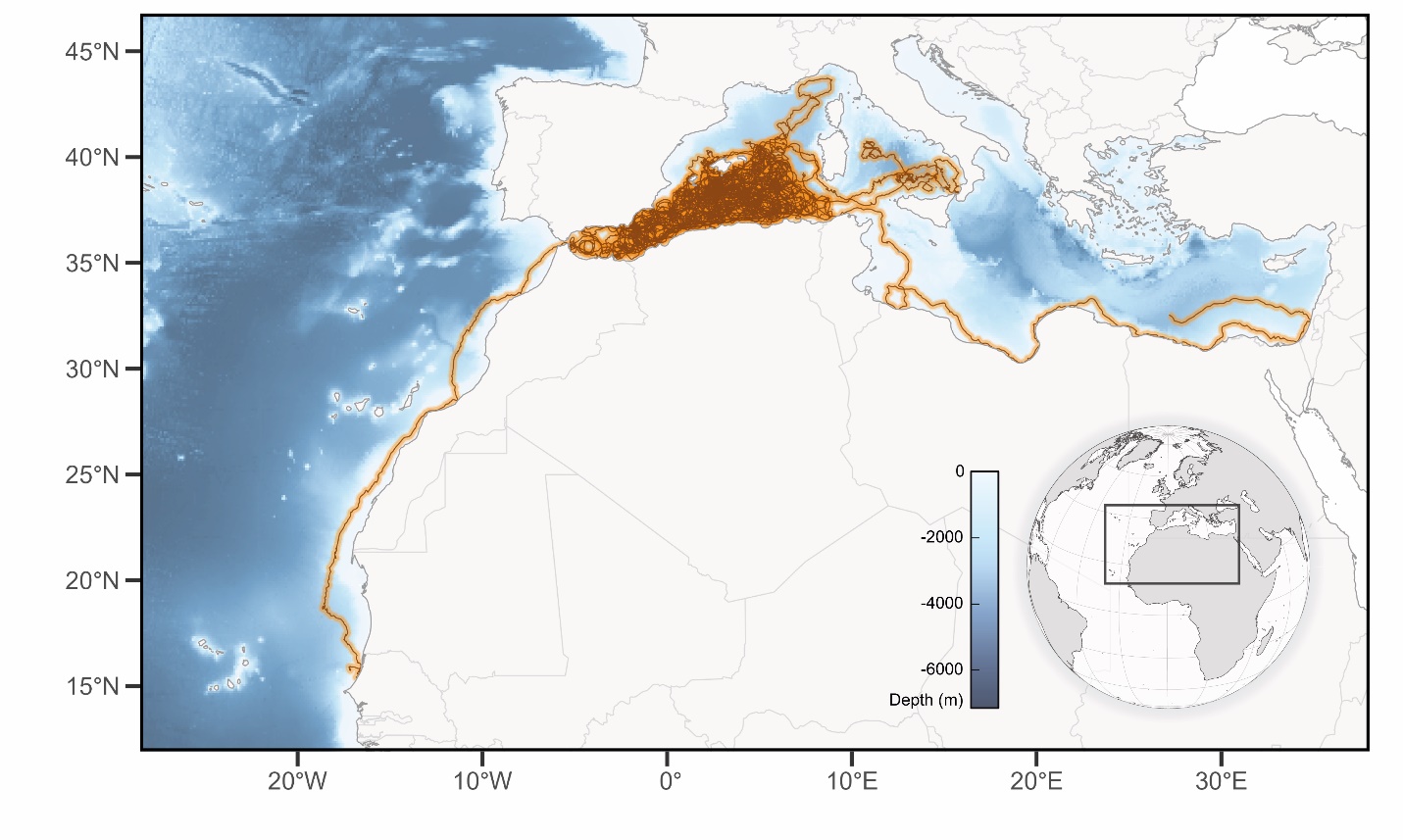


Supplementary Figure 1. Study area and individual trajectories (i.e. state-space-model predictions) for all the loggerhead turtles (n = 28) tagged for this study in the Western-Mediterranean.


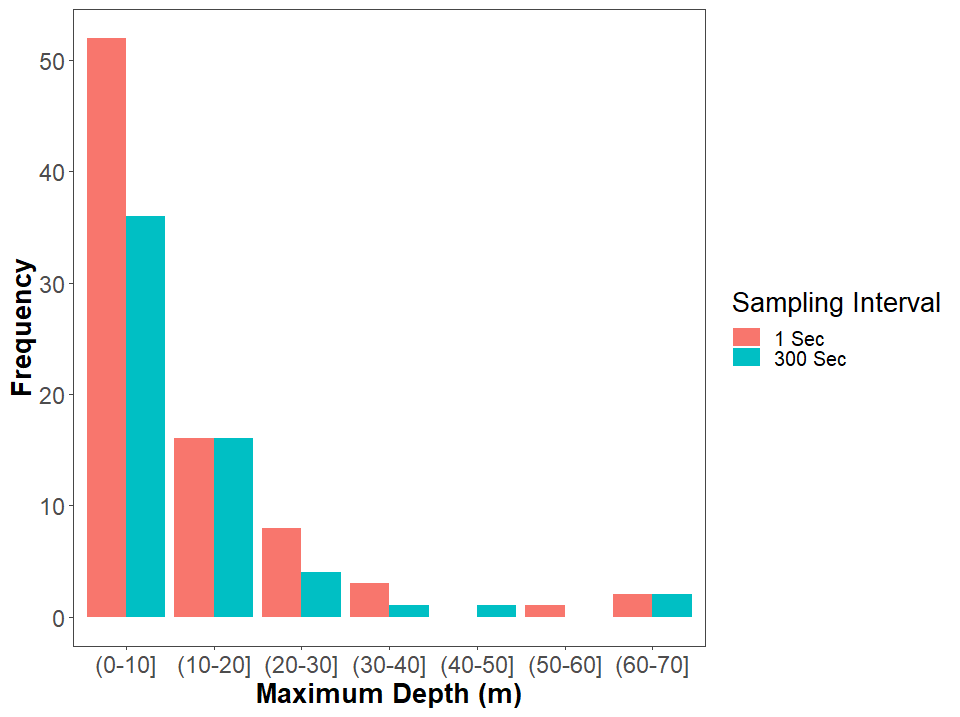

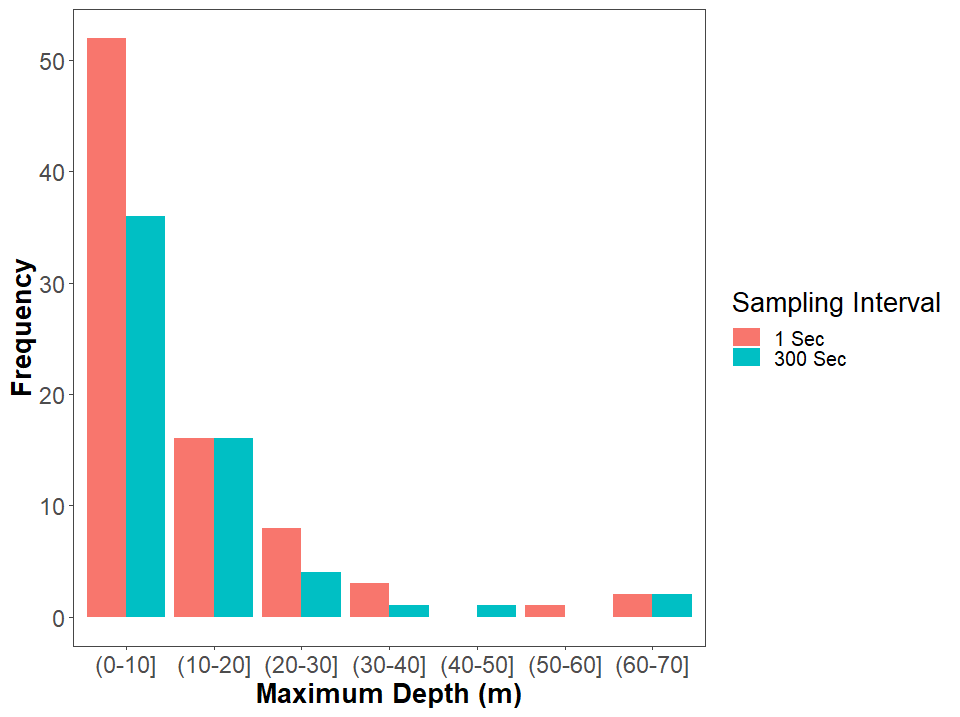


Supplementary Figure 2. Validation of the TDR processing method. Frequency of dives with binned maximum depths at both 1 second and 300 second resampled data. No significant difference in frequency was identified for dives with a maximum depth greater than 10m. Data correspond to a single loggerhead turtle equipped with a Survivorship PAT (sPAT, Wildlife Computers).


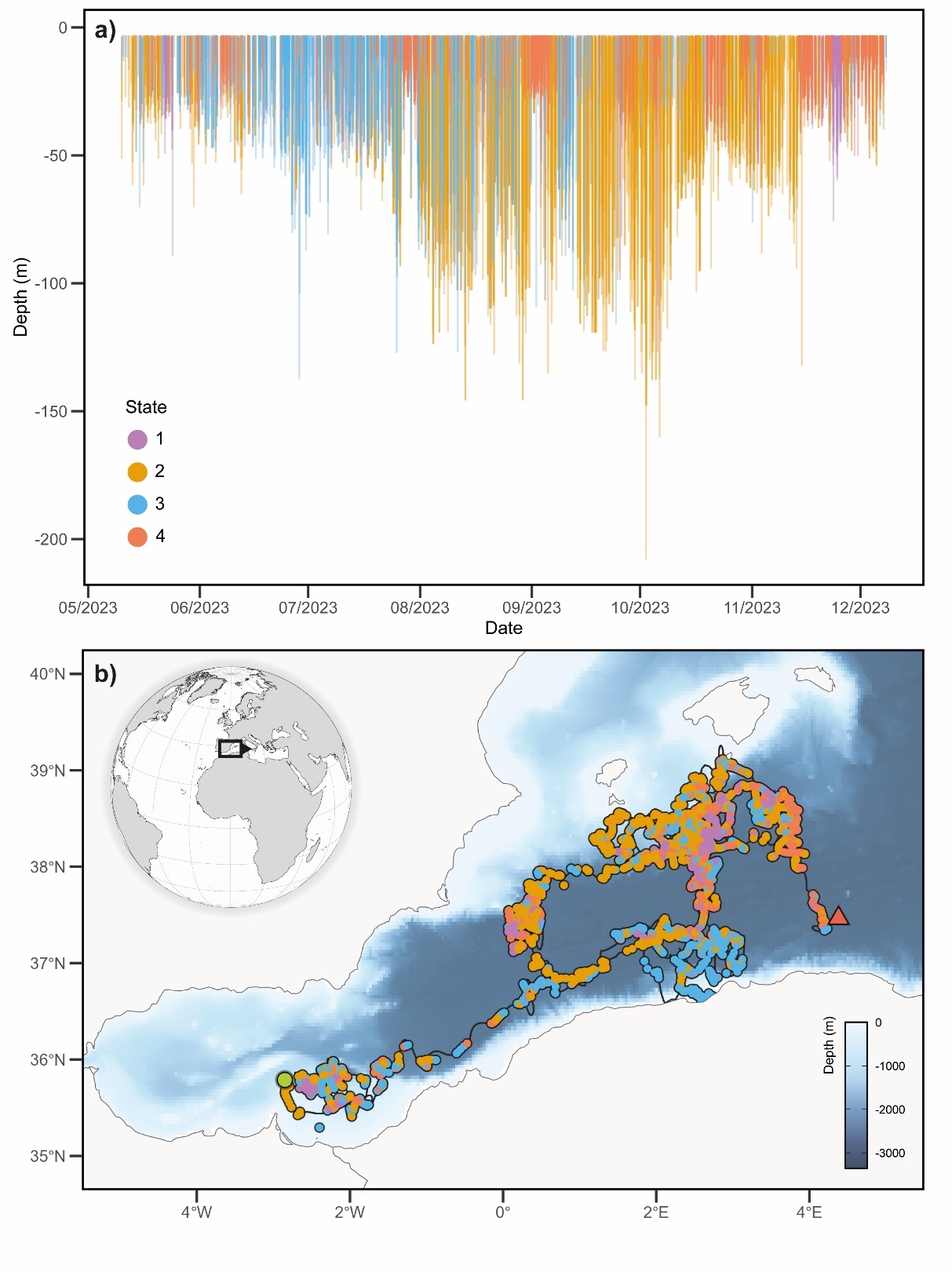


Supplementary Figure 3. Dive profile of a single TDR deployment (organism ID 235396) used in this study. (**a**) TDR data spanning 214 days, depicting behavioural states over time. State 1= Purple, State 2= Orange, State 3= Blue and State 4= Coral. (**b**) Location of each behavioural state throughout deployment; Green dot and red triangle showing the beginning and the ending of the track visualized, respectively.


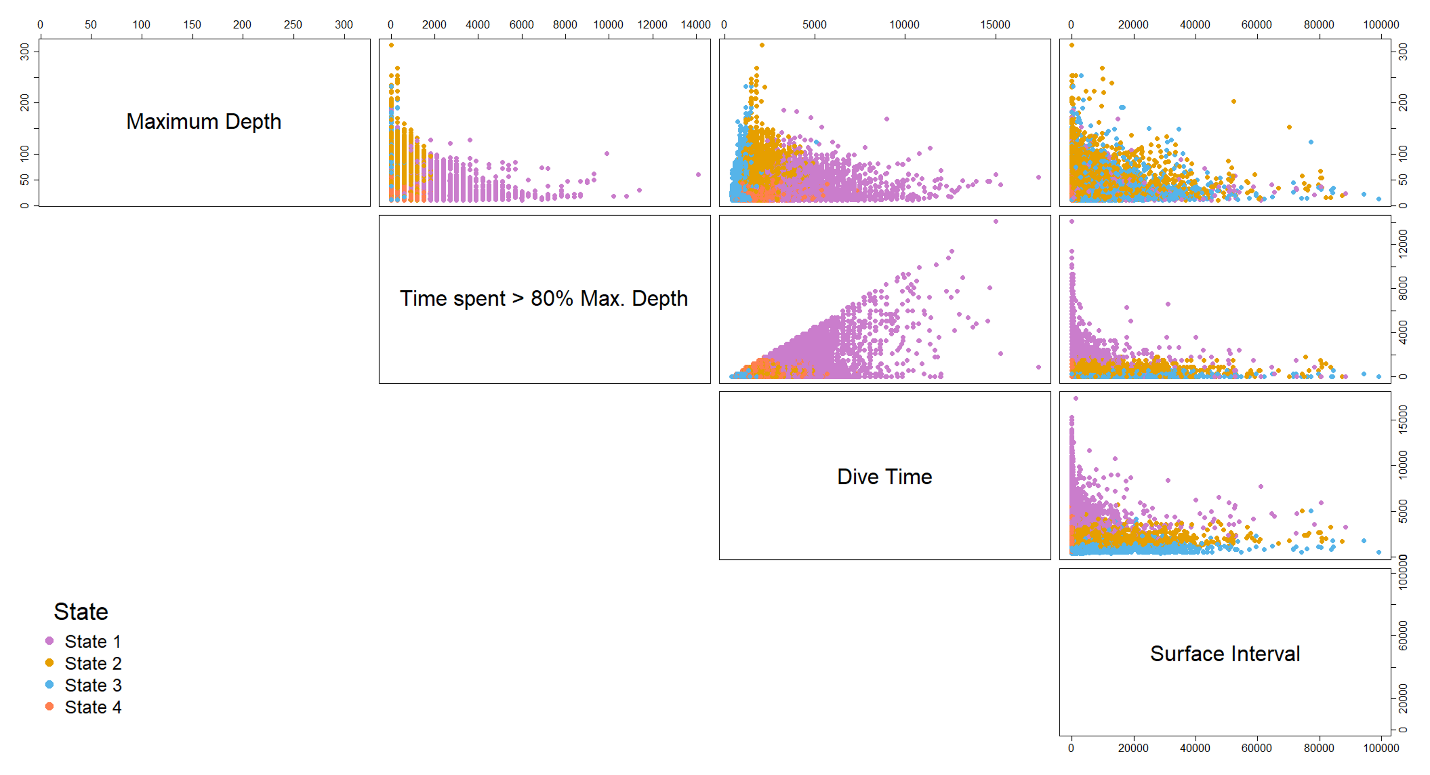


Supplementary Figure 4. Scatterplot matrix for values of each variable used in the HMM for 42,618 dives across the four identified states.


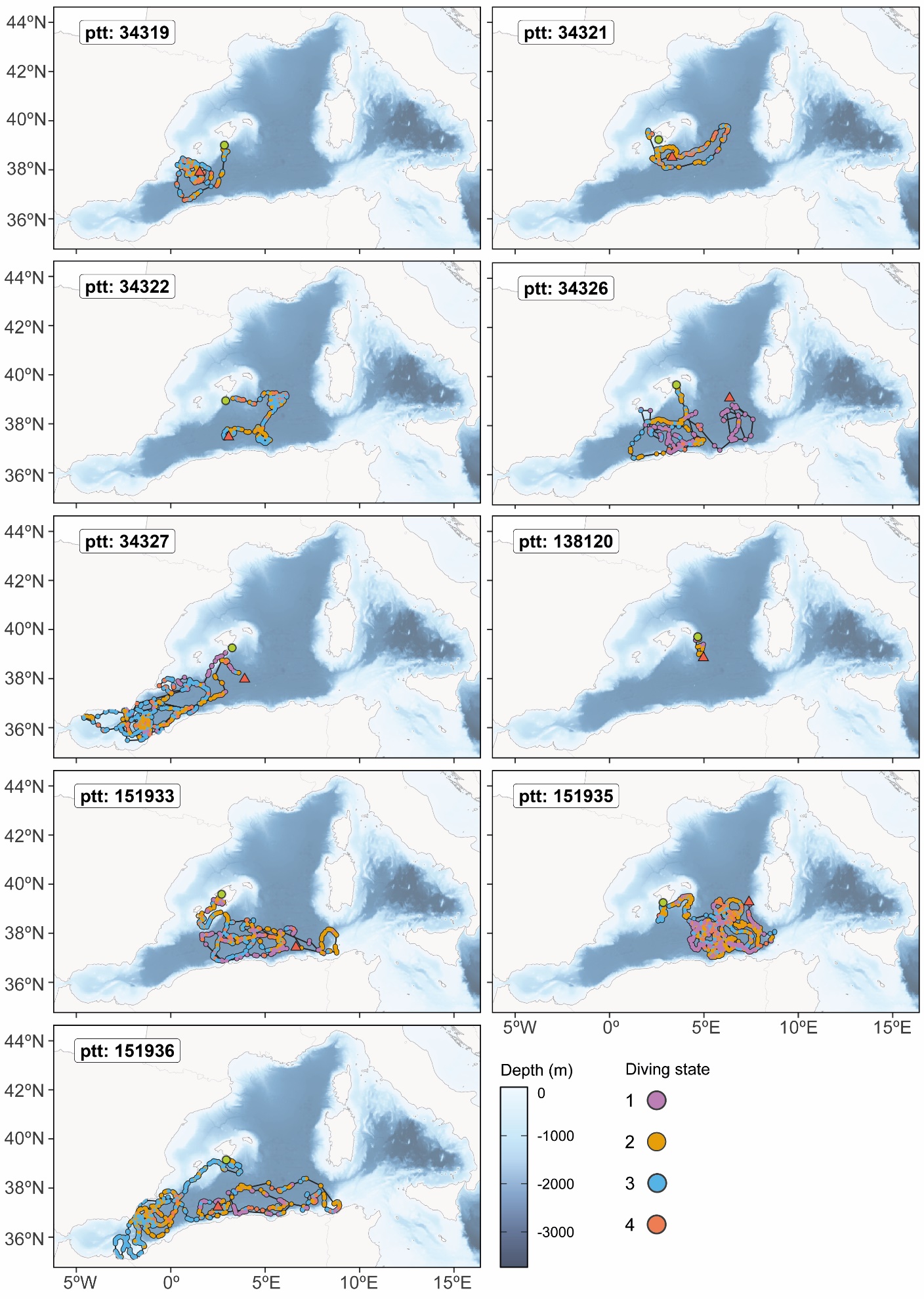

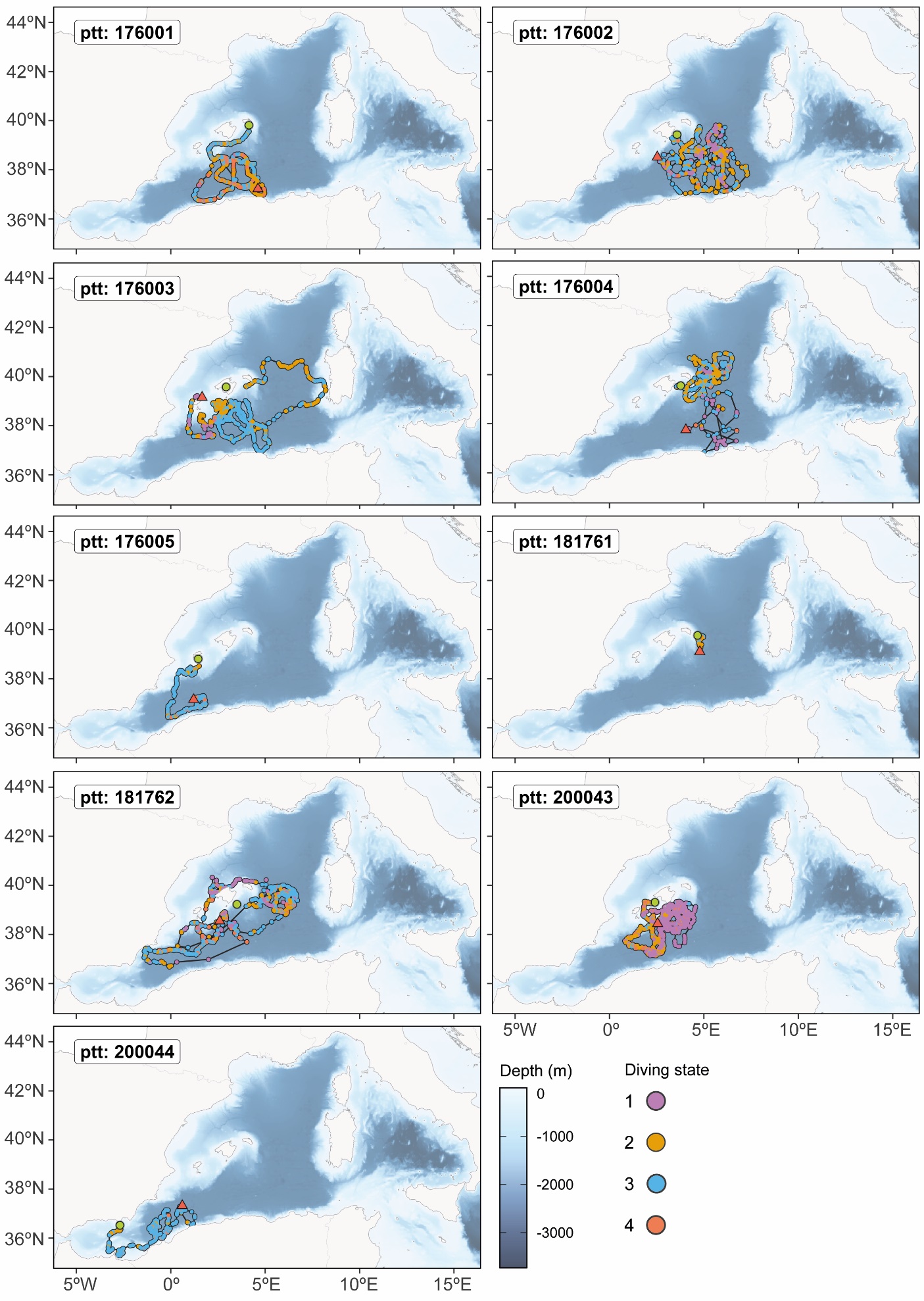

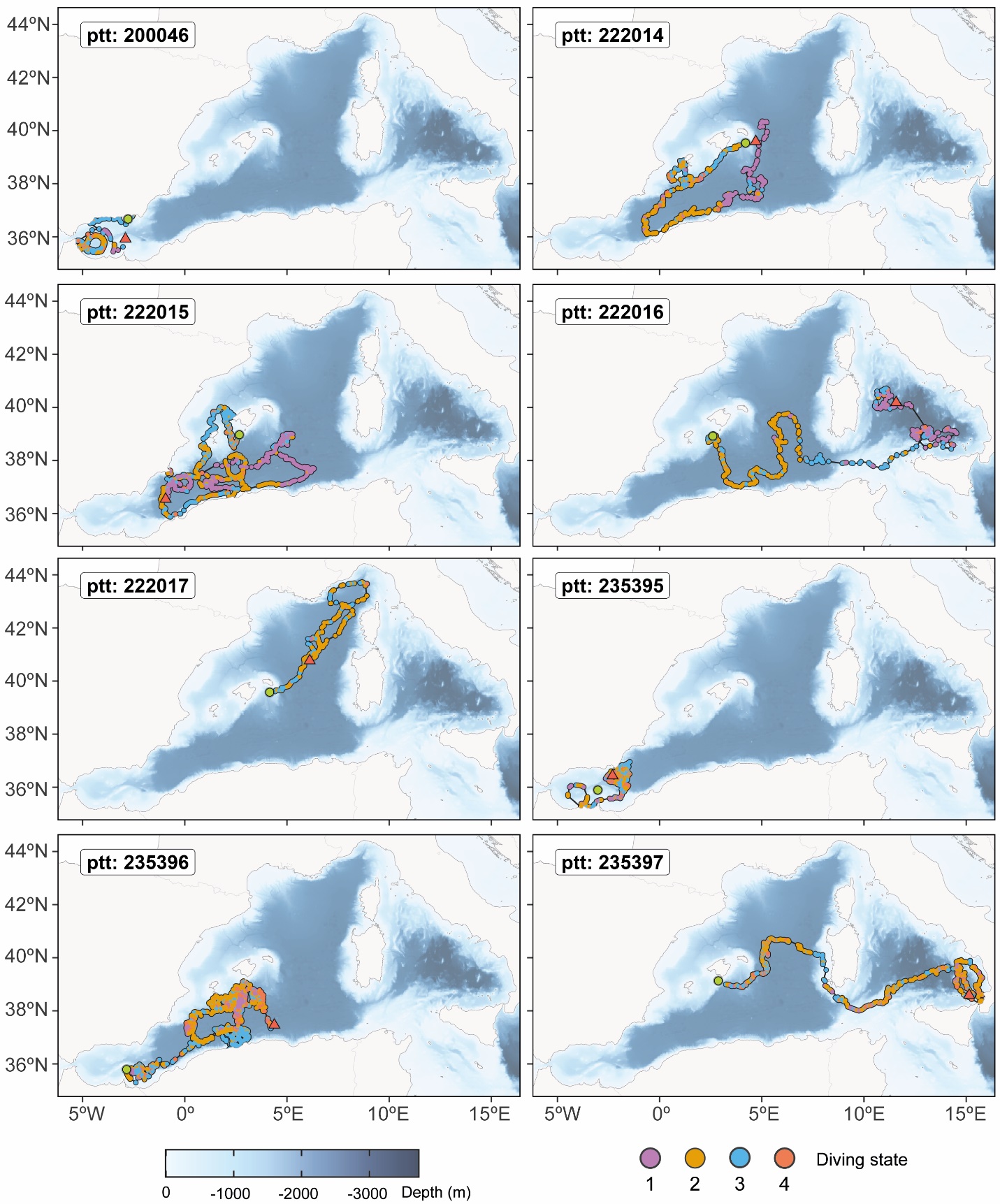

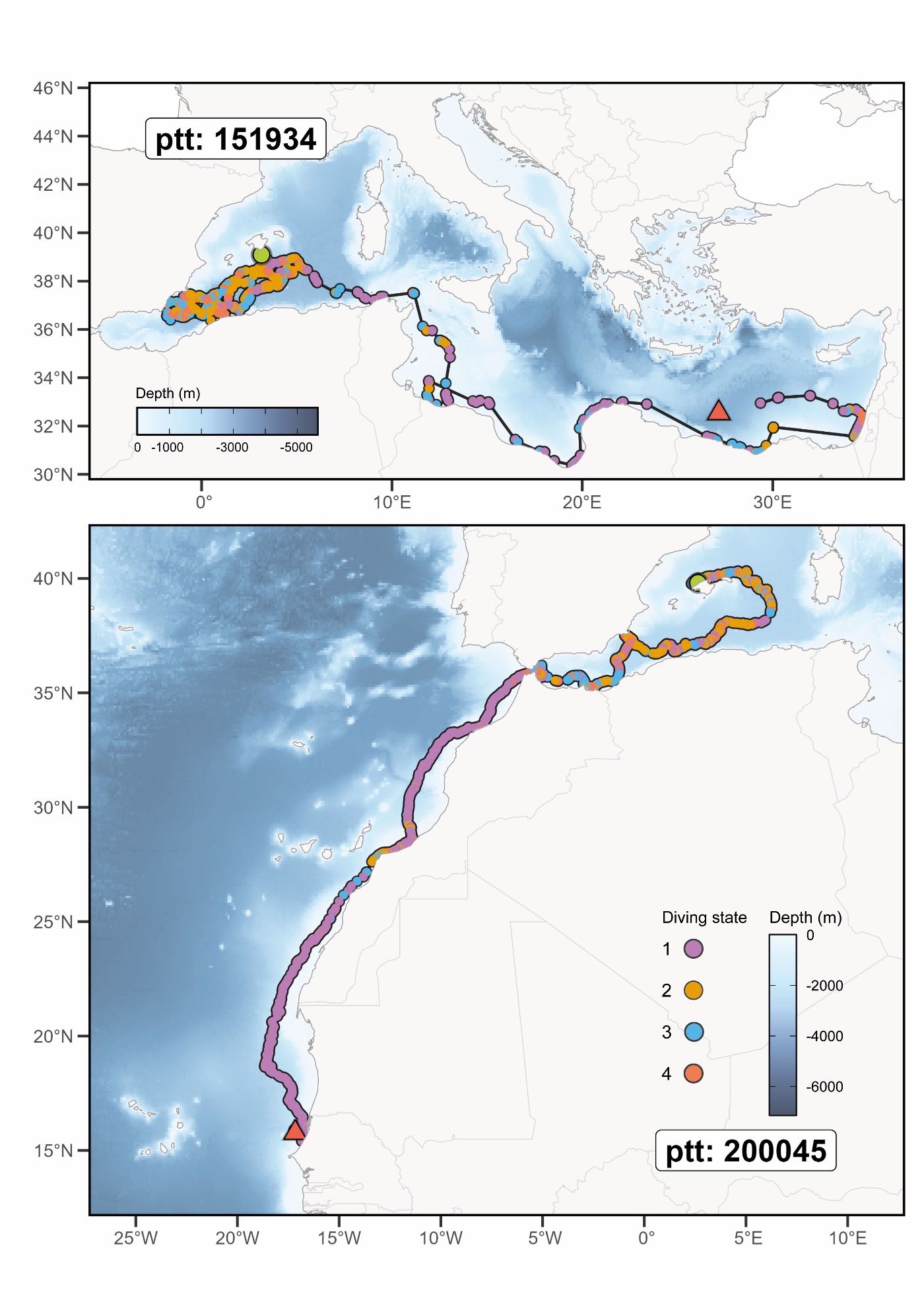


Supplementary Figure 5. Location of the different dives and their diving states identified for each tagged sea turtle (ppt as Organism ID) along State Space Model predicted tracks. The green circle and red triangle indicate the first and last recorded positions, respectively .


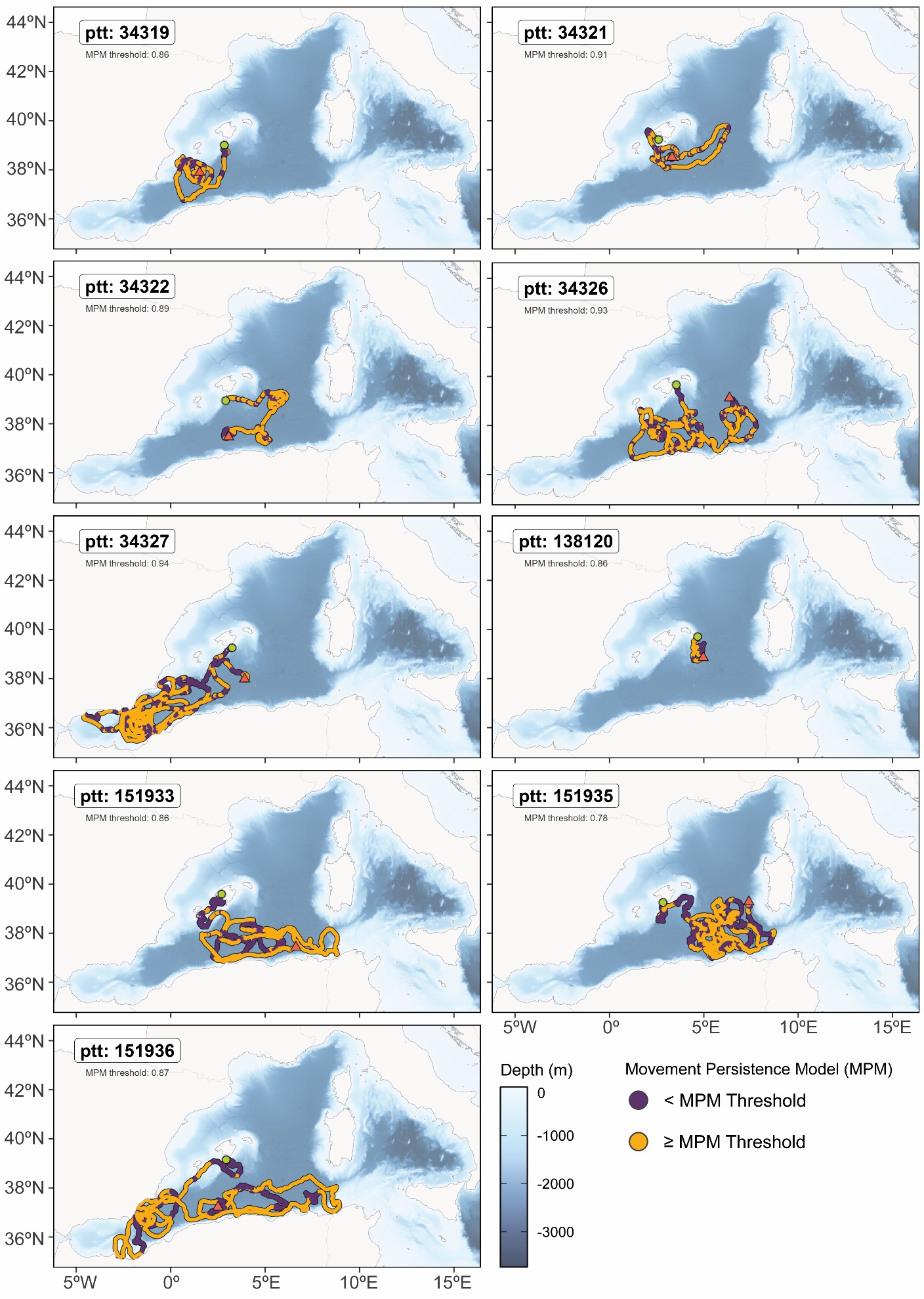

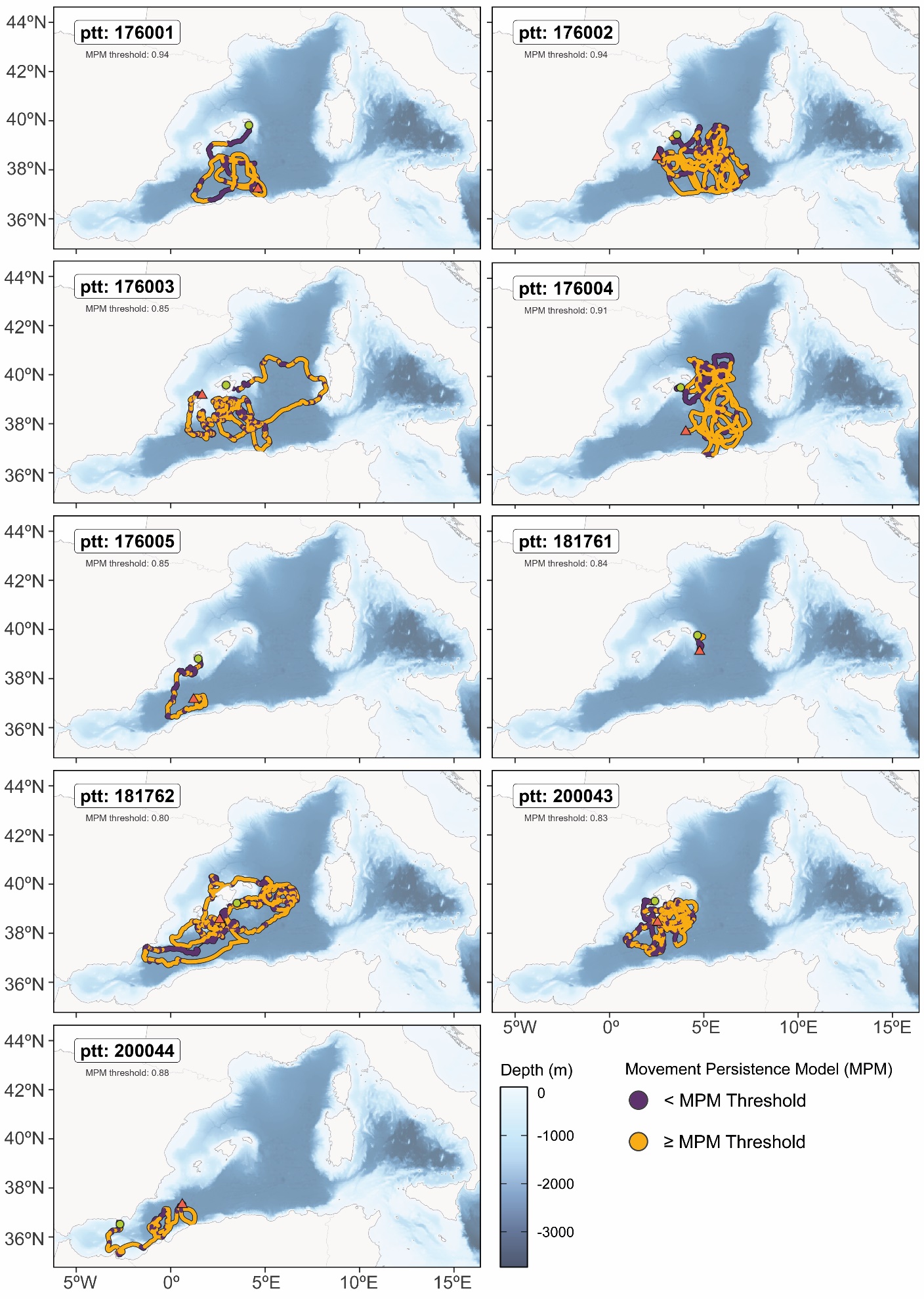

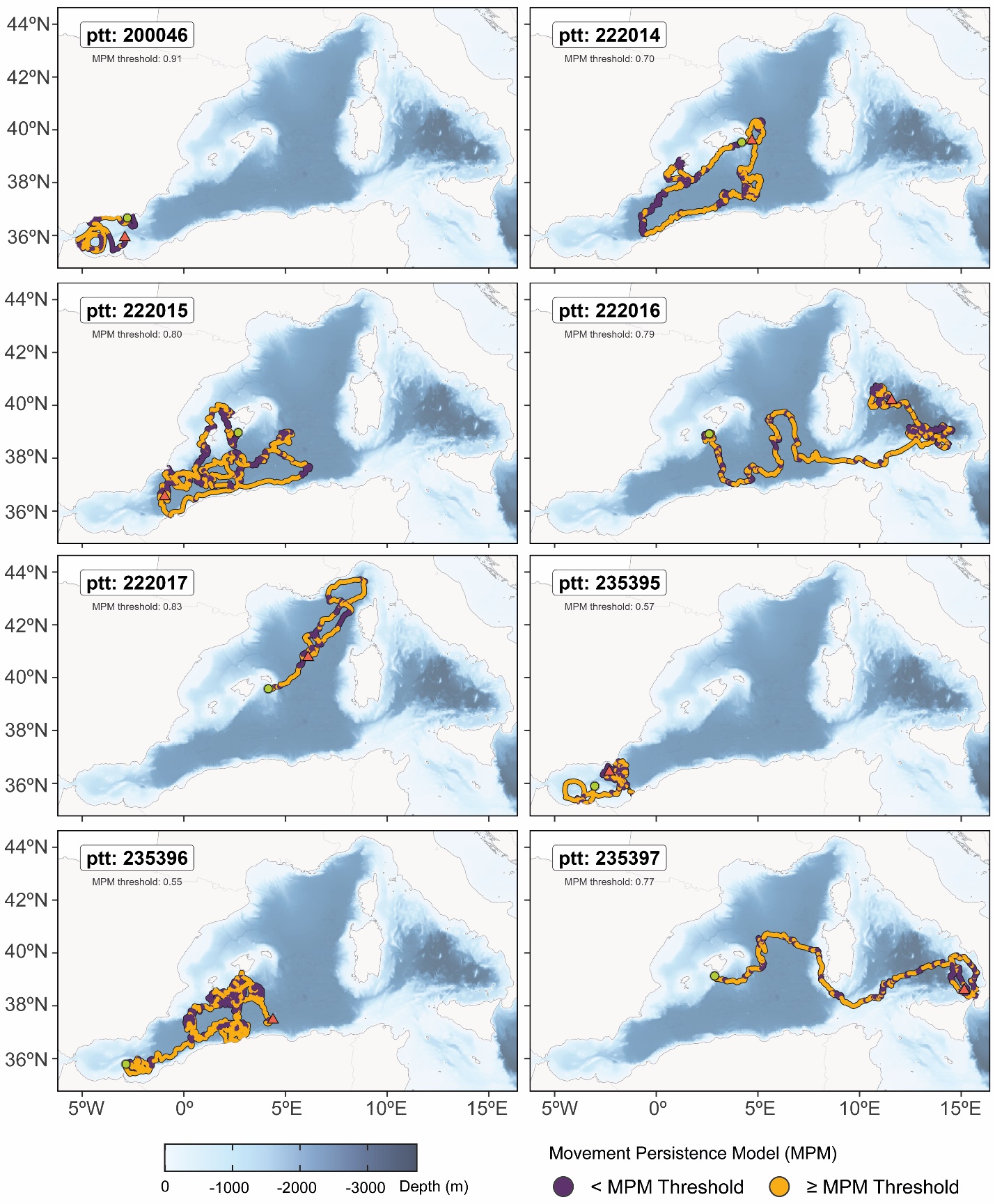

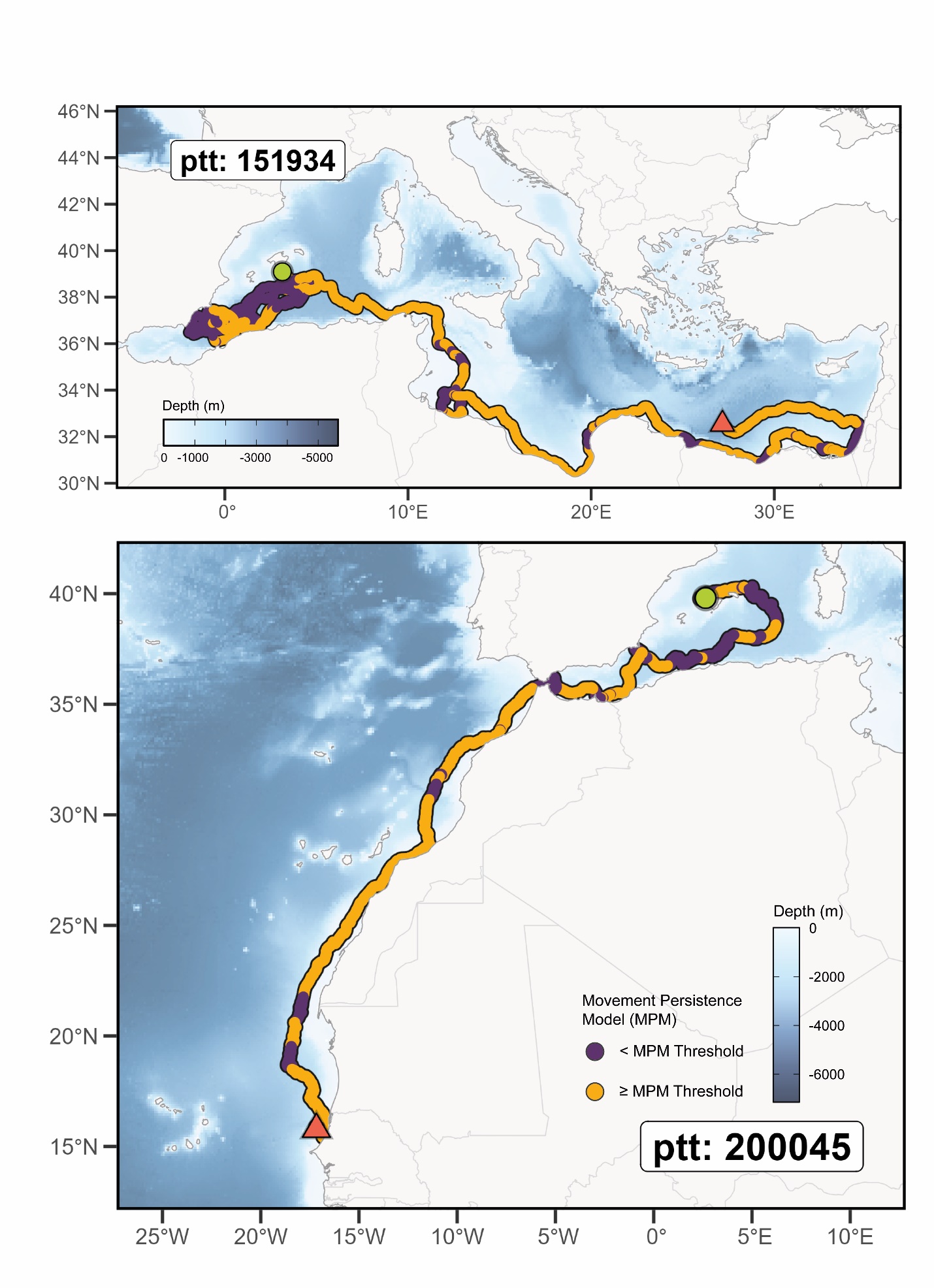


Supplementary Figure 6. Binary classification of the move persistence index (*g*) (Movement Persistence Model, MPM) for each tagged sea turtle (*ppt* as Organism ID) along the different State Space Model predicted locations. The MPM threshold relative for each individual suppose the median of *g* value of all its positions. The green circle and red triangle indicate the first and last registered positions, respectively.
